# A Rare Ocular Manifestation of Lipoid Proteinosis

**DOI:** 10.7759/cureus.82423

**Published:** 2025-04-17

**Authors:** Xin Gen Ng, Jessica MPT, Ee Ling Ang

**Affiliations:** 1 Department of Ophthalmology, Hospital Pulau Pinang, George Town, MYS

**Keywords:** autosomal recessive disorders, extracellular matrix protein, hyaline, lipoid proteinosis (lp), moniliform blepharosis

## Abstract

Lipoid proteinosis (LP) is a rare genetic disorder passed down in an autosomal recessive pattern. It affects multiple body systems and is marked by the abnormal buildup of hyaline material in different tissues. These deposits lead to changes in the oral mucosa and skin, beaded eyelid lesions, hoarseness of voice, and neurological symptoms. This case report describes a 20-year-old woman with an eight-year history of abnormal skin lesions on her eyelids. Examination revealed continuous yellow-white, beaded lesions along the upper and lower eyelid margins of both eyes. Her visual acuity was 6/9 bilaterally, with an intraocular pressure of 14 mmHg and normal anterior segment findings. Fundus examination was unremarkable. Systemic manifestations included waxy skin thickening, papular lesions on the knees, elbows, and back, and alopecia. She also exhibited thickened oral mucosa and oral stomatitis. Additionally, she presented with a hoarse voice caused by vocal cord nodules, delayed developmental milestones, and suspected memory loss. A skin biopsy confirmed the presence of hyaline material consistent with LP. She is currently receiving multidisciplinary care involving a dermatologist, otorhinolaryngologist, neurologist, and ophthalmologist. This case highlights the important role ophthalmologists play in the diagnosis of LP. Given the pathognomonic ocular feature - moniliform blepharosis - ophthalmologists may serve as key contributors to early detection, facilitating timely interdisciplinary collaboration. A multidisciplinary approach can help prevent complications and improve the patient’s quality of life.

## Introduction

Lipoid proteinosis (LP) is a genetic disorder inherited in an autosomal recessive pattern. It is a systemic condition characterized by abnormal hyaline deposition in the mucous membranes, skin, central nervous system, and other parts of the body [1]. LP is caused by mutations in the extracellular matrix protein 1 gene located on chromosome 1q21 [2,3]. Both males and females are affected equally [2]. Although cases have been reported worldwide, the disease is more prevalent in the Namaqualand region of South Africa [2].

Moniliform blepharosis is considered a pathognomonic ocular manifestation of LP [4]. The condition typically presents in early childhood with hoarseness and severe dysphonia, followed by cutaneous abnormalities, as well as neurologic and ocular involvement [1].

## Case presentation

**Figure 1:**
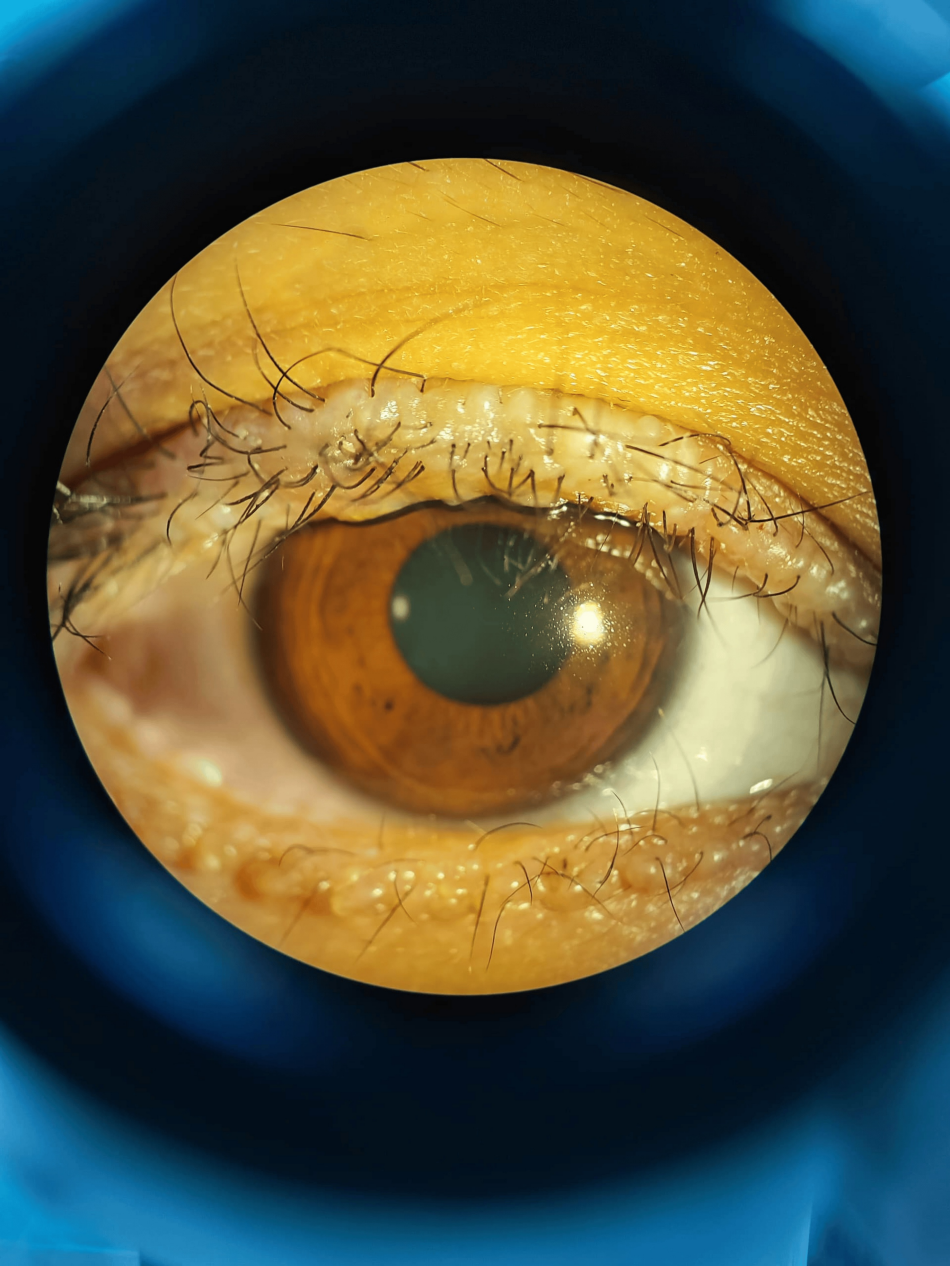
Moniliform blepharosis affecting the right upper and lower eyelids

**Figure 2:**
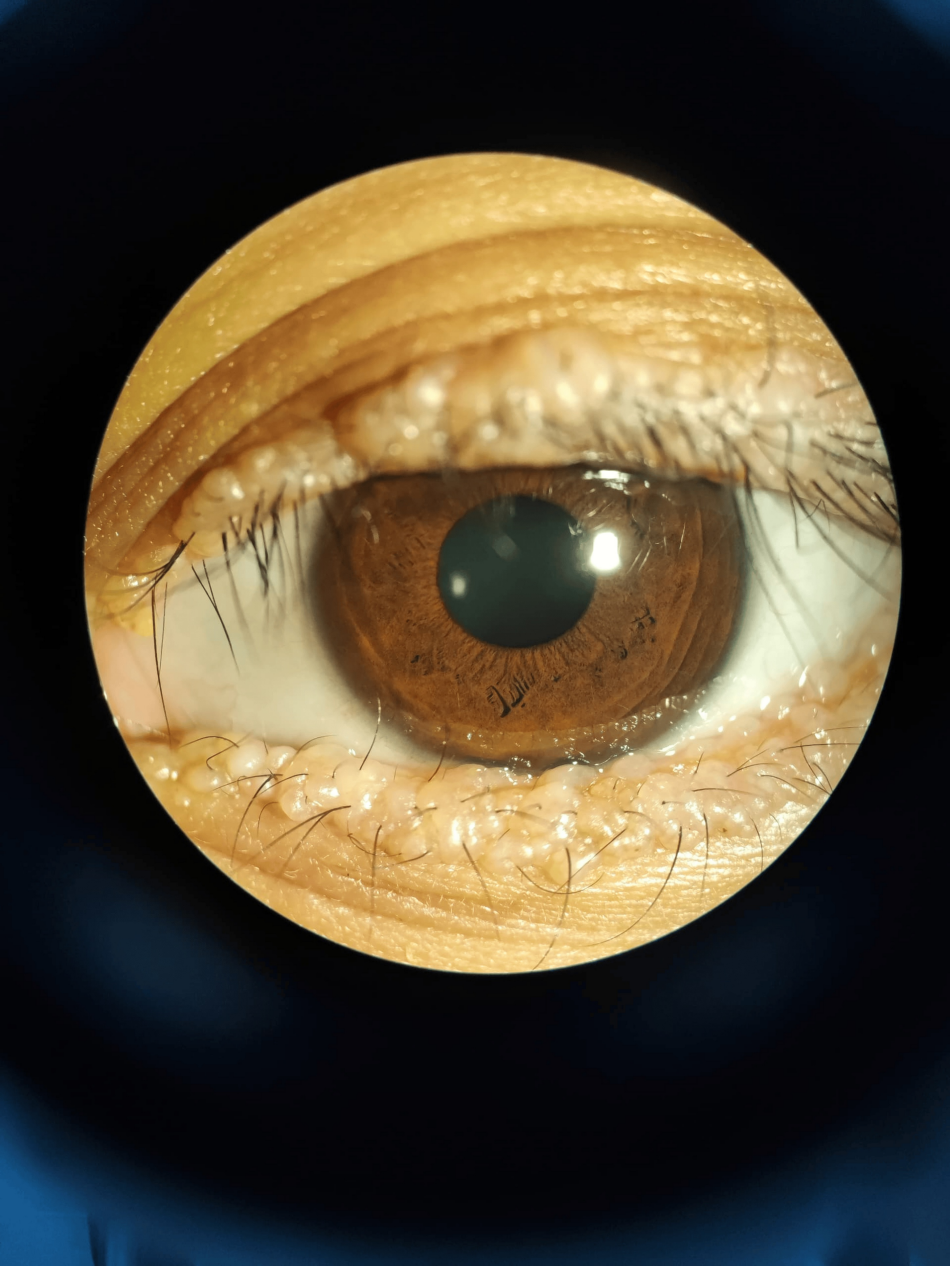
Moniliform blepharosis affecting the left upper and lower eyelids

A 20-year-old woman presented with an eight-year history of abnormal skin lesions on her eyelids. Examination revealed continuous yellow-white beaded lesions along the upper and lower eyelid margins of both eyes (Figure 1, Figure 2). Visual acuity was 6/9 bilaterally, with an intraocular pressure of 14 mmHg and normal anterior segment findings, including a tear break-up time of more than 10 seconds. Fundus examination was unremarkable.

**Figure 3:**
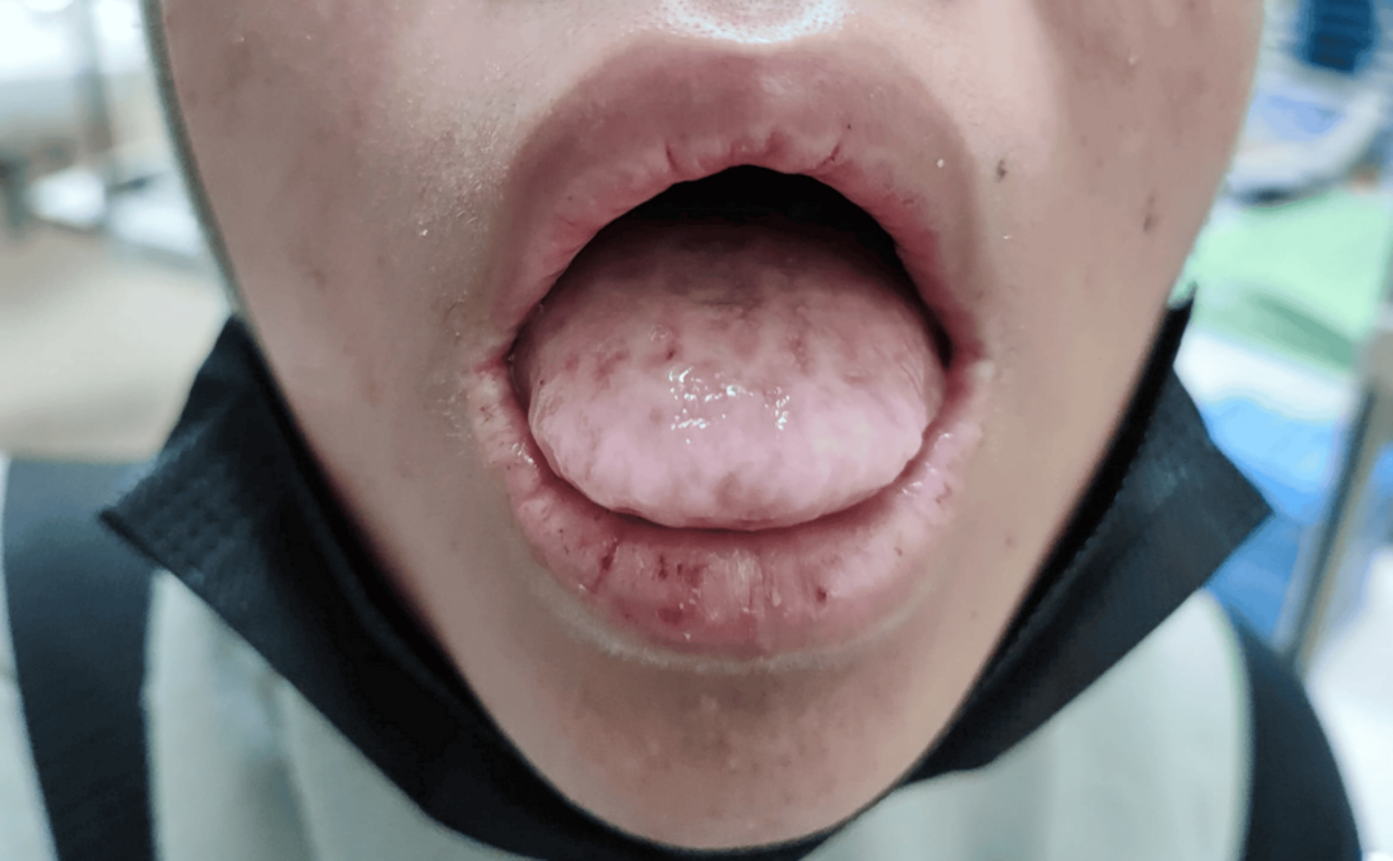
Woody tongue with thickened oral mucosa

Systemic features included cutaneous lesions with waxy skin thickening, papules on the knees, elbows, and back, as well as alopecia. She also had thickened oral mucosa and oral stomia (Figure 3). Additionally, she exhibited hoarseness of voice due to a vocal cord nodule, delayed developmental milestones, and suspected memory loss.

On further history-taking, the patient reported that her parents and siblings did not exhibit similar symptoms, although she was uncertain about her grandparents and other relatives. She was initially diagnosed by the dermatology team with pemphigus vulgaris due to the presentation of skin lesions, oral mucosal involvement, and hoarseness of voice, which may mimic LP.

Upon referral to our team for ocular screening, the patient did not exhibit any ocular signs typically associated with pemphigus vulgaris, such as conjunctivitis, keratinization, or cicatricial changes. The only notable finding was the presence of beaded lesions along the upper and lower eyelid margins bilaterally. Following this, she was referred back to the dermatology team, who then decided to perform a skin biopsy of the eyelid.

**Figure 4:**
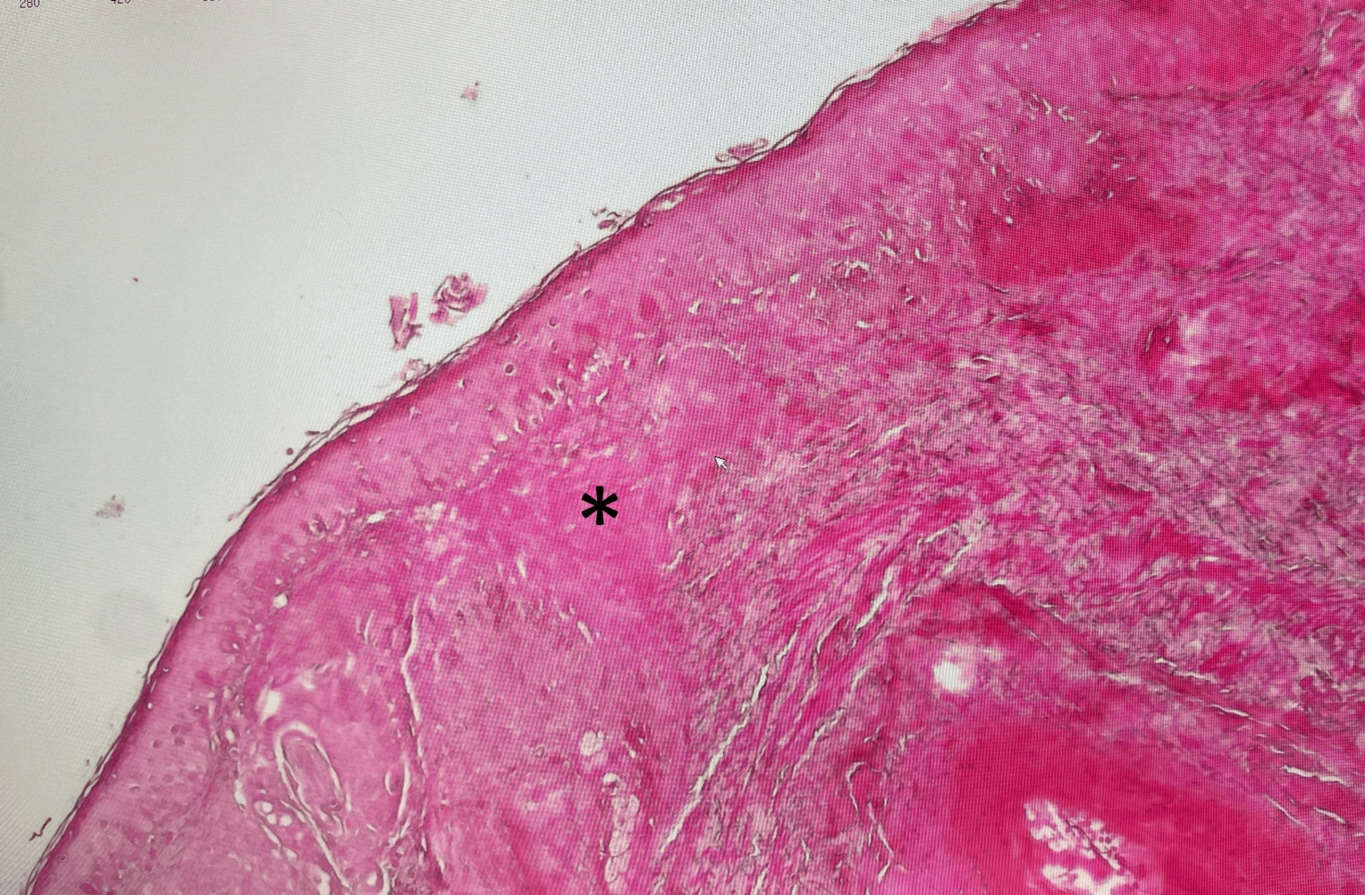
Eyelid skin biopsy showing extensive hyaline material (asterisk) deposited within the dermis The hyaline appears as eosinophilic, homogeneous deposits on H&E staining (original magnification ×10).

The biopsy revealed multiple deposits of amorphous, eosinophilic, acellular hyaline material within the superficial dermis (Figure 4). The overlying epidermis showed flattened rete pegs. The surrounding dermis displayed infiltration by a few lymphocytes and histiocytes, with mild focal pigmentary incontinence. No malignancy was observed.

Histopathological findings were consistent with LP. The deposits stained positive with periodic acid-Schiff (PAS) and PAS with diastase. Congo red staining for amyloid was negative.

Subsequently, the diagnosis was revised to LP. The patient is currently under multidisciplinary care, including a dermatologist, otorhinolaryngologist, neurologist, and ophthalmologist. Our ophthalmology team has provided conservative treatment for the patient, with annual follow-ups.

## Discussion

LP, also known as Urbach-Wiethe syndrome, is a rare condition that occurs worldwide. It was first described by Urbach and Wiethe in 1929 [5]. Males and females are affected equally, but the condition has a higher incidence in countries where consanguinity is more common [1].

A pathognomonic ocular finding for LP is beaded lesions along the eyelid margin, known as moniliform blepharosis, which occurs in approximately 50% of patients [6]. As noted by Ali et al. [7], these lesions may obstruct the punctum, leading to epiphora. Additionally, the lacrimal glands can be infiltrated with hyaline material, causing dry eyes. Other reported ocular associations include open-angle glaucoma, drusen in the macula, retinitis pigmentosa, uveitis, and cataracts, as described by Abtahi et al. [8]. Aside from moniliform blepharosis, no other ocular findings were observed in this patient.

LP typically presents with hoarseness and severe dysphonia, characterized by a husky voice that persists throughout life due to hyaline deposits on the vocal cords [9]. Cutaneous lesions generally appear initially as vesicles or bullae with hemorrhagic crusts around the face and mouth [10]. Additionally, hyaline deposits in the skin can lead to generalized skin thickening, as well as the development of waxy, yellow papules and nodules on various parts of the body [9]. Several neurological abnormalities can result from hyaline deposition in the central nervous system, and patients may present with epilepsy [9]. Calcification of the hippocampus-amygdala complex may further contribute to neuropsychiatric manifestations, including cognitive abnormalities, memory loss, and hallucinations [9,11].

Histologically, LP displays hyaline-like deposits that stain positive for PAS and resist diastase. These deposits are predominantly found in the dermis and along the dermal-epidermal junction. Immunolabeling is a valuable diagnostic tool, particularly in the early stages of the disease. Additionally, Congo red staining helps differentiate LP from amyloidosis, which is often part of the differential diagnosis [1].

The patient described in this case report presented with most of the ocular and systemic manifestations as described. Multidisciplinary management is essential for addressing the diverse manifestations of LP. It is rare for ophthalmologists to be the first to diagnose LP, and it is often described as being delayed and difficult [12]. Early recognition of its ocular features can aid in the timely diagnosis and management of this complex condition.

## Conclusions

This report emphasizes the crucial role of ophthalmologists in diagnosing LP. Given the pathognomonic ocular feature of moniliform blepharosis, ophthalmologists can be key contributors to early disease detection. Recognizing these characteristic eyelid lesions can prompt timely interdisciplinary collaboration, ensuring comprehensive patient care. Early referral to dermatologists, otorhinolaryngologists, neurologists, and psychiatrists is essential for systemic assessment and initiating appropriate management. A multidisciplinary approach can help prevent complications and significantly improve the patient’s quality of life.
